# A Glycosaminoglycan-Rich Fraction from Sea Cucumber *Isostichopus badionotus* Has Potent Anti-Inflammatory Properties In Vitro and In Vivo

**DOI:** 10.3390/nu12061698

**Published:** 2020-06-06

**Authors:** Leticia Olivera-Castillo, George Grant, Nuvia Kantún-Moreno, Hirian A. Barrera-Pérez, Jorge Montero, Miguel A. Olvera-Novoa, Leydi M. Carrillo-Cocom, Juan J. Acevedo, Cesar Puerto-Castillo, Victor May Solís, Juan A. Pérez-Vega, Judit Gil-Zamorano, Enrique Hernández-Garibay, María A. Fernández-Herrera, Mayra Pérez-Tapia, Oscar Medina-Contreras, Jairo R. Villanueva-Toledo, Rossanna Rodriguez-Canul, Alberto Dávalos

**Affiliations:** 1 Departamento Recursos del Mar, Centro de Investigación y de Estudios Avanzados del IPN-Unidad Mérida, Antigua Carretera a Progreso Km. 6, Merida 97310, Yucatan, Mexico; nuviakm@gmail.com (N.K.-M.); lostrinosdeldiablo@gmail.com (J.M.); miguel.olvera@cinvestav.mx (M.A.O.-N.); cesarp@cinvestav.mx (C.P.-C.); victormay_29@hotmail.com (V.M.S.); juan.vega@cinvestav.mx (J.A.P.-V.); rossana.rodriguez@cinvestav.mx (R.R.-C.); 2 School of Medicine, Medical Sciences and Nutrition, University of Aberdeen, Aberdeen AB25 2ZD, UK; g.grant@abdn.ac.uk; 3 Applied Science Research Foundation, A.C., Calle 26 No. 144 x 21 y 21A, Col. San Pedro Cholul, Merida 97138, Yucatan, Mexico; 4 Laboratorio de Anatomía Patológica (ANAPAT), Av. Yucatán 630, Fracc. Jardines de Mérida, Mérida 97135, Yucatan, Mexico; hbarrerap@gmail.com; 5 Facultad de Ingeniería Química, Universidad Autónoma de Yucatán, Calle 43 No. 613 x 90, Col. Inalámbrica, Mérida 97069, Yucatan, Mexico; leydi.carrillo@correo.uady.mx; 6 Departamento de Fisiología y Farmacología, Facultad de Medicina, Universidad Autónoma del Estado de Morelos, Calle Leñeros s/n, Col. Los Volcanes, Cuernavaca 62350, Morelos, Mexico; juan.acevedo@uaem.mx; 7 IMDEA Food Institute, CEI UAM+CSIC, Carretera de Cantoblanco 8, 28049 Madrid, Spain; judit.gil@imdea.org; 8 Centro Regional de Investigación Pesquera de Ensenada, Carretera Tijuana-Ensenada Km. 107.5, El Sauzal de Rodríguez, Ensenada 22860, Baja California, Mexico; enrique.garibay@inapesca.gob.mx; 9 Departamento de Física Aplicada, Centro de Investigación y de Estudios Avanzados del IPN-Unidad Mérida, Antigua Carretera a Progreso Km. 6, Mérida 97310, Yucatan, Mexico; marietafernandezh@gmail.com; 10 Instituto Politécnico Nacional, Unidad de Investigación, Desarrollo e Innovación Médica y Biotecnológica (UDIMEB), Prolongación de Carpio y Plan de Ayala s/n, Col. Santo Tomás, Del. Miguel Hidalgo, Ciudad de México 11340, Mexico; smpt.2011@hotmail.com; 11 Immunology and Proteomics Laboratory, Mexico Children’s Hospital “Federico Gómez”, Mexico City 06720, Mexico; omedina@himfg.edu.mx; 12 Cátedras CONACYT-Fundacion IMSS, A.C., CONACYT, Av. Insurgentes Sur 1582, Del. Benito Juárez, Col. Crédito Constructor, Ciudad de Mexico 03940, Mexico; jvillanuevat@conacyt.mx

**Keywords:** holothuroids, glycosaminoglycans, inflammation, ear-inflammation

## Abstract

Sea cucumber body wall contains several naturally occurring bioactive components that possess health-promoting properties. *Isostichopus badionotus* from Yucatan, Mexico is heavily fished, but little is known about its bioactive constituents. We previously established that *I. badionotus* meal had potent anti-inflammatory properties in vivo. We have now screened some of its constituents for anti-inflammatory activity in vitro. Glycosaminoglycan and soluble protein preparations reduced 12-O-tetradecanoylphorbol-13-acetate (TPA)-induced inflammatory responses in HaCaT cells while an ethanol extract had a limited effect. The primary glycosaminoglycan (fucosylated chondroitin sulfate; FCS) was purified and tested for anti-inflammatory activity in vivo. FCS modulated the expression of critical genes, including NF-ĸB, TNFα, iNOS, and COX-2, and attenuated inflammation and tissue damage caused by TPA in a mouse ear inflammation model. It also mitigated colonic colitis caused in mice by dextran sodium sulfate. FCS from *I. badionotus* of the Yucatan Peninsula thus had strong anti-inflammatory properties in vivo.

## 1. Introduction

Sea cucumbers are used as food supplements or traditional medicines in many parts of the world. In Asia, they are ascribed a wide range of health-promoting actions, including antimicrobial, anti-thrombotic, anti-coagulant, neuroprotective, wound healing, antioxidant, anti-hypertensive, anti-tumor, anti-inflammatory, and immune-stimulating properties [1,2]. Harvested species belong to genera including *Apostichopus*, *Atinopyga*, *Cucumaria*, *Holothuria*, *Isostichopus*, *Parastichopus* and *Stichopus*. These are known to contain many potential therapeutic compounds, such as triterpene glycosides (saponins), fucosylated chondroitin sulfates, fucoidans, glycosaminoglycans, sulfated polysaccharides, sterols, phenolics, cerebrosides, lectins and proteins/peptides [1,2]. However, it remains unclear exactly which compounds or combinations thereof are required to reliably treat or manage specific diseases. Further, the profile and concentrations of the naturally occurring bioactive components in sea cucumbers is species-, locality-, and growth environment-dependent. Recent work has therefore concentrated on isolation and characterization of individual constituent bioactive compounds from sea cucumber and assessment of their specific health-modulating properties in vitro and in vivo [1,2,3].

*Isostichopus badionotus* is distributed along the coast of the Caribbean Sea and Western Atlantic Ocean. In recent years, the species has been intensively harvested in Mexican waters, particularly around the Yucatan Peninsula [4,5]. Remarkably, despite the high demand for this sea cucumber species, little is known of its nutritional, medicinal, or therapeutic properties in vivo. Two studies have established that *I. badionotus* from the Yucatan Peninsula has potential health-promoting effects. Diets containing the *I. badionotus* body wall were hypo-cholesterolaemic for rats [6]. In addition, they substantially altered gene expression in the rat intestine by down-regulating pro-inflammatory genes and up-regulating genes essential for gut barrier integrity and repair [7]. Further, studies in other in vitro and in vivo models confirmed the anti-inflammatory properties of *I. badionotus* body wall meal [7].

The present research aimed to identify anti-inflammatory factors from the body wall of *I. badionotus* from the Yucatan Peninsula and assess their efficacy in vivo.

## 2. Materials and Methods

### 2.1. Sea Cucumber Collection and Processing

Adult *Isostichopus badionotus* (Selenka, 1867) were collected from the seafloor off the coast of Sisal, Yucatan, Mexico (SAGARPA permit No. DGOPA/1009/210809/08761) and processed as before to obtain desalted, lyophilized sea cucumber meal [6].

### 2.2. Preliminary Extractions

A crude ethanol extract was obtained from desalted and lyophilized sea cucumber meal following Guo et al. [8], a soluble protein extract was prepared according to Ridzwan et al. [9] and a crude glycosaminoglycan preparation (GAGs) prepared using the basic technique of Vieira et al. [10]. The compositions of these preliminary extracts were monitored by thin-layer chromatography or polyacrylamide gel electrophoresis.

### 2.3. Large-Scale Extraction and Characterization of Glycosaminoglycans (GAGs)

GAGs were isolated essentially according to Vieira et al. [10]. Ten grams (10 g) desalted and lyophilized meal was added to 300 mL 0.1 M sodium acetate buffer (pH 6) containing 5 mM EDTA, 5 mM L-cysteine and 1 g papain. The mixture was incubated at 60 °C for 24 h, and the resulting enzymatic liquor centrifuged (2000× *g* 10 min at 10 °C). The supernatant was mixed with two volumes 95% ethanol, stored overnight at −10 °C and the precipitate recovered by centrifugation. This was resuspended and dialyzed against distilled water in a 12–14 kDa cut-off membrane (Spectra/Por). The preparation was further fractionated by preparative anion exchange chromatography on a QXL Hitrap column (GE Healthcare) fitted to an Akta Pryme plus (GE Healthcare). Elution was done with NaCl (up to 1.2 M made up in 20 mM Tris-HCl, pH 8.0) and absorbance (280 nm) and conductivity monitored.

### 2.4. Chemical Characterization of GAGs Fraction

Total carbohydrates were determined by UV spectrophotometry based on a method using sulfuric acid to form furfural derivatives [11]. Uronic acids were quantified using meta-hydroxy diphenyl with galacturonic acid as a standard [12]. Sulfates were measured using potassium sulfate as a standard [13]. Total proteins were determined with the bicinchoninic acid assay using a commercial kit (BCA Protein Assay kit, Thermo Scientific). Fucose was quantified using deoxy sugar and L-fucose as standards [14].

Gram-negative and Gram-positive bacteria, fungi and yeasts were screened for by culturing the GAGs preparations on appropriate agar plates or liquid media. Assessment of lipopolysaccharides (LPS) content was done using a commercial kit (Pierce LAL Chromogenic Endotoxin Quantitation, Thermo Scientific; detection limit <1 EU/mL) following manufacturer instructions. No viable microbes or LPS were detected in the GAGs samples.

### 2.5. Infrared Spectroscopy

Spectra were recorded on an Agilent Cary 630 FTIR spectrometer within the wavenumber range between 4000–600 cm^−1^, using the Attenuated total reflection (ATR) technique. The standards employed for the IR analysis were L-fucose, D-glucuronic acid, D (+) galacturonic acid, and chondroitin sulfate from shark.

### 2.6. NMR Spectroscopy 

The 1H NMR spectra of crude GAGs samples were measured at 25 °C and 600 MHz using an Agilent spectrometer. For the purified GAGs the spectra were measured at 400 MHz using a Bruker Advance 400 Ultrashield apparatus. Spectra processing was done with the MestReNova software (Mestrelab Research, Santiago de Compostela, Spain).

### 2.7. Polyacrylamide Gel Electrophoresis 

PAGE was performed in a 6% polyacrylamide gel (37.5% acrylamide/1% bisacrylamide) using a mini-protean system (Bio-Rad, Mexico City, Mexico). Briefly, GAGs in Tris-HCL buffer (pH 6.8) were mixed with non-reducing sample buffer, boiled for 5 min, loaded onto the gel and electrophoresed at 100 V for 110 min. The gel was stained with 0.5% Alcian Blue solution in 3% acetic acid/25% isopropanol for two hours and de-stained with a 10% acetic acid/40% ethanol solution overnight. Finally, the gel was digitized. A broad range molecular weight standard (Bio-Rad, Mexico City, Mexico) was used. 

### 2.8. Action of GAGs on TPA-Stimulated HaCaT (Human Keratinocyte) Cells and Mouse Splenocytes In Vitro

Spontaneously immortalized human keratinocytes (HaCaT) were cultured in Dulbecco’s Modified Eagle Medium (DMEM) supplemented with 10% fetal bovine serum (FBS). 3 × 10^5^ HaCat cells were seeded in 96-well plates (Corning Costar Sigma Mexico) and cultured overnight until 80% confluence. Media was replaced and cells were stimulated with 3.2 mg/mL GAGs sample in the presence of 2.5 ng/mL TPA [15]. After 24 h media were collected, and the cells were re-stimulated with 2.5 ng/mL 12-O-tetradecanoylphorbol-13-acetate (TPA) and 5 µg/mL brefeldin for 3 h. The cells were harvested with 0.25% trypsin/1 mM EDTA and stained for flow cytometry analysis.

Isolated HaCaT/splenocyte cells were resuspended in phosphate buffered saline (PBS) containing 5% FBS. Unspecific staining was blocked with PBS-10% FBS for 15 min at 4 °C. Cells were permeabilized using fixation/permeabilization buffer (eBioscience) for 15 min. Samples were then stained at 4 °C for 30 min with AlexaFluor 647-labeled IFNγ antibodies, then washed two times in permeabilization/wash buffer. An amount of 3 × 10^5^ events were acquired on a CytoFlex (Beckman Coulter, Brea, CA, USA) flow cytometer, and analyzed using the CytExpert software (Beckman Coulter) [16]. 

### 2.9. Action of GAGs on the Viability of Fibroblasts In Vitro

A thiazolyl blue tetrazolium blue (MTT) assay with human fibroblast cell line (hFB) was used to evaluate the viability of GAGs activity [17]. Cells were cultivated routinely in DMEM/F-12 medium without phenol red and supplemented with 10% FBS at 37 °C in a 5% CO^2^ and humidified atmosphere. Cells were seeded at 2 × 10^4^ cells/well in 100 μL medium into 96-well microplates and incubated for 24 h. Fresh culture medium (100 μL) containing GAGs was then added to the wells to final concentrations of 0.039, 0.078, 0.156, 0.313, 0.625 and 1.25 mg/mL. Cells exposed only to culture medium were used as a control, and cells exposed only to medium without cells were used as a background. Cells with dimethyl sulfoxide (DMSO) were used as a toxicity control, and cells with recombinant human fibroblast growth factor (rFGF) were used as a proliferation control. After 48 h incubation, 100 µL MTT (5 mg/mL) were added to each well and left for 4 h. The medium was then removed and 200 µL DMSO was added to resuspend the formazan crystals. Optical density (OD) was measured at 570 nm by a spectrophotometric plate reader (Multiskan FC Thermo Scientific). The percentage of relative cell viability was calculated as:

[(OD treated − OD background)/(OD control − OD background)] × 100%

Data were expressed as the mean ± standard deviation and analysed with a one-way analysis of variance (ANOVA) test followed by Tukey’s post hoc test (*p* < 0.05). Analysis was run on the GraphPad Prism 7 statistical program.

### 2.10. Experimental Animals

Male CD1 and C57Bl/6 mice were obtained from the Centre for Research and Advanced Studies of the National Polytechnic Institute (CIVNESTAV-IPN) at Zacatenco, Mexico. Animal experiments were performed at CINVESTAV-IPN Mérida. Animals were kept under standard conditions (12 h light/dark cycle, 23 ± 2 °C, 65% humidity). Food and water were freely available. Animals were acclimatized for seven days prior to the experiment. All experimental protocols were approved by the Institutional Animal Care and Use Committee of CINVESTAV-IPN (No. 0126-15) and complied with the applicable Mexican Official Norm (NOM-062-ZOO-1999), “Technical Specifications for the Care and Use of Laboratory Animals”, as well as all applicable federal and institutional regulations.

### 2.11. Mouse Ear Anti-Inflammatory Activity

Anti-inflammatory activity was evaluated using the mouse ear model, as described previously [7,18]. Under anesthesia (pentobarbital 25 mg/kg animal weight), acute inflammation was induced by topical application of 12-O-tetradecanoylphorbol-13-acetate (TPA; phorbol myristate acetate; Sigma Cat. P8139; 2.5 µg in 10 µL acetone) to both surfaces of the right ear of CD1 mice. Ten microliters (10 µL) vehicle were topically applied to each surface of the left ear. GAGs extract (125 µg/20 µL water/per ear, each surface) or the anti-inflammatory dexamethasone (DXA) were applied (250 µg/10 µL water/per ear, each surface) 15 min after treatment with TPA. Six hours later, the mice were re-anesthetized, and an electronic micrometer used to assess ear edema and thickness. The animals were then euthanized by cervical dislocation, and each ear removed and weighed (total ear weight). A sterile biopsy punch (Integra Miltex^®^, Integra LifeSciences Corp, PA.USA.) was used to remove two representative samples (5 mm diameter discs) from each ear, and the discs weighed. Inflammation was quantified as the difference between the average weight of the right ear discs (TPA alone or TPA+dexamethasone or TPA+extract) and that of the left ear discs (vehicle: acetone). Samples were taken from the ear discs for analysis of gene expression (immersed in 400 µL RNAlater [Ambion] and stored at −80 °C) and for histological evaluation (fixed in 10% formalin).

### 2.12. Histological Analysis of Ear Samples

Fixed samples were mounted in paraffin blocks (Richard-Allan Scientific Paraffin Type 6^®^, ThermoFisher Scientific, Mexico City, Mexico, cut into sections (2 µm thickness) with a ThermoScientific Microm HM 325^®^ rotary microtome (Mexico City, Mexico) and processed using an AutotechniconDuo^®^ 2A System(Technicon Instruments Corp, New York, NY, USA). Sections were stained simultaneously with haematoxylin-eosin (H&E), examined with a conventional optical microscope and images taken with a digital camera (Evolution™ LC Color; Olympus, Mexico City, Mexico). A representative area was selected for qualitative light microscopic analysis. Histology scores for ear samples were generated by two independent pathologists and averaged for analysis. Briefly, samples were evaluated for the presence of neutrophil infiltration and edema, and each factor deemed to be either null, slight, moderate or intense [6,7]. Numerical values were assigned to each intensity: null (0); slight (1); moderate (2); and intense (3). A sample’s total score was calculated by adding the values assigned each factor.

### 2.13. Dextran Sodium Sulfate (DSS)-Colitis

Sixteen C57Bl/6 mice (8 weeks old, 25 g) were given free access to water containing Dextran Sodium Sulfate (MP Biomedicals, USA, DSS, 36–50 kDa) [25 g/liter] for 4 days and water for a further 2 days. In addition, half of the mice were dosed orally with GAGs solution (2 mg/mouse/d; ~80mg/kg body weight (BW)/d) on days −1, 0, and +1 to +5 while the remainder were daily administered a vehicle substance. The mice were euthanized by cervical dislocation on day 6 and dissected. The small intestine and colon were removed, their length measured, and representative samples of colon collected for analysis. Untreated control mice had free access to water throughout the study.

### 2.14. Gene Expression

RNA was isolated from tissue using the Animal Tissue RNA Purification Kit (Norgen), following manufacturer instructions. Genomic DNA (gDNA) was removed using the TURBO DNA-free™ Kit (Ambion). cDNA was obtained using the RevertAid H Minus First Strand cDNA kit (Thermo Scientific), according to manufacturer instructions. Single-strand DNA (ssDNA) was diluted at a 1:20 ratio for further use in real-time PCR (qRT-PCR). Quantitative real-time PCR (qRT-PCR) assays were run using specific oligos in a Rotor Gene Q (2-plex) real-time PCR detection system with QuantiNova SYBR Green PCR master mix (Qiagen). Reactions (including non-template as negative controls) were performed in quadruplicate. Thermo-cycling conditions were 5 min at 95 °C, followed by 40 cycles of 15 s at 94 °C, and 45 s at 62 °C (annealing-extension). β-actin (ACTB) [ear assay] and elongation factor 2 (EEF2) [DSS-colitis] were used as housekeeping genes. Relative expression was calculated using the 2^−∆∆Ct^ method, as previously described [19], and results expressed relative to the controls. 

### 2.15. Statistical Analysis

Statistical analysis was applied using generalized linear modelling (GLM) to estimate the effect of the factor levels (i.e., ear-weight results), as well as the variable of gene expression (fold change) for genes. Over-dispersion was detected (from residual Poisson regression model) and corrected using Poisson GLM. The standard errors were multiplied by the square root of the dispersion parameter using a quasi-Poisson GLM model. The variance; Var [Yi] = φµ, where μ is the mean and φ the dispersion parameter, was calculated with the GLM model. Significant difference was set at *p*-value < 0.05. A customized R (www.r-project.org) function and GraphPad Prism were used for statistical analyses.

## 3. Results

### 3.1. Extracts from the Body-Wall of I. badionotus Inhibit TPA-Induced Proliferation of Keratinocyte and Splenocyte Cells in Culture

Previous data from our group indicated that sea cucumber *I. badionotus* meal had potent anti-inflammatory activity in vitro and in vivo [7]. To ascertain the general nature of the naturally occurring bioactive components in *I. badionotus* responsible for this ameliorative action, an initial study was conducted in which three extracts of desalted and lyophilized sea cucumber meal (an ethanolic extract [8], a soluble protein extract [9] and a crude glycosaminoglycan (GAGs) preparation [10]) were prepared and assayed for their effects on TPA-stimulated human keratinocyte (HaCaT) cells and mouse splenocytes in vitro.

**Figure 1:**
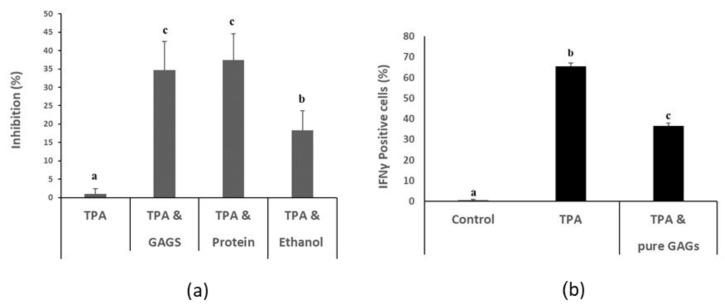
Extracts (crude GAGs, soluble proteins, ethanol soluble) from the body wall of *I. badionotus* inhibit 12-O-tetradecanoylphorbol-13-acetate (TPA)-induced proliferation of HaCat [human keratinocyte] cells in culture (**a**). Purified GAGs (3 μg/mL) limit TPA-mediated production of Interferon gamma (IFNγ)-positive HaCaT cells (**b**). N ≥ 4 per group and values with distinct superscripts differ significantly from each other (*p* ≤ 0.05).

The GAGs and soluble protein fractions prepared from *I. badionotus* body wall inhibited TPA-mediated proliferation of HaCat cells (Figure 1a) and of mouse splenocytes (Supplemental Figure S1a). In contrast, while the ethanol extract, expected to contain saponins, partially reduced TPA-induced proliferation of HaCaT cells (Figure 1a), it had limited activity against TPA-stimulated splenocytes (Supplemental Figure S1a). Analysis of the preliminary extracts revealed that the GAGs fraction was comprised of primarily one major component whereas both the protein and ethanol extracts were complex mixtures (data not shown). The present studies therefore concentrated on the GAGs fraction. 

### 3.2. Large-Scale Preparation and Characterization of GAGs

**Figure 2:**
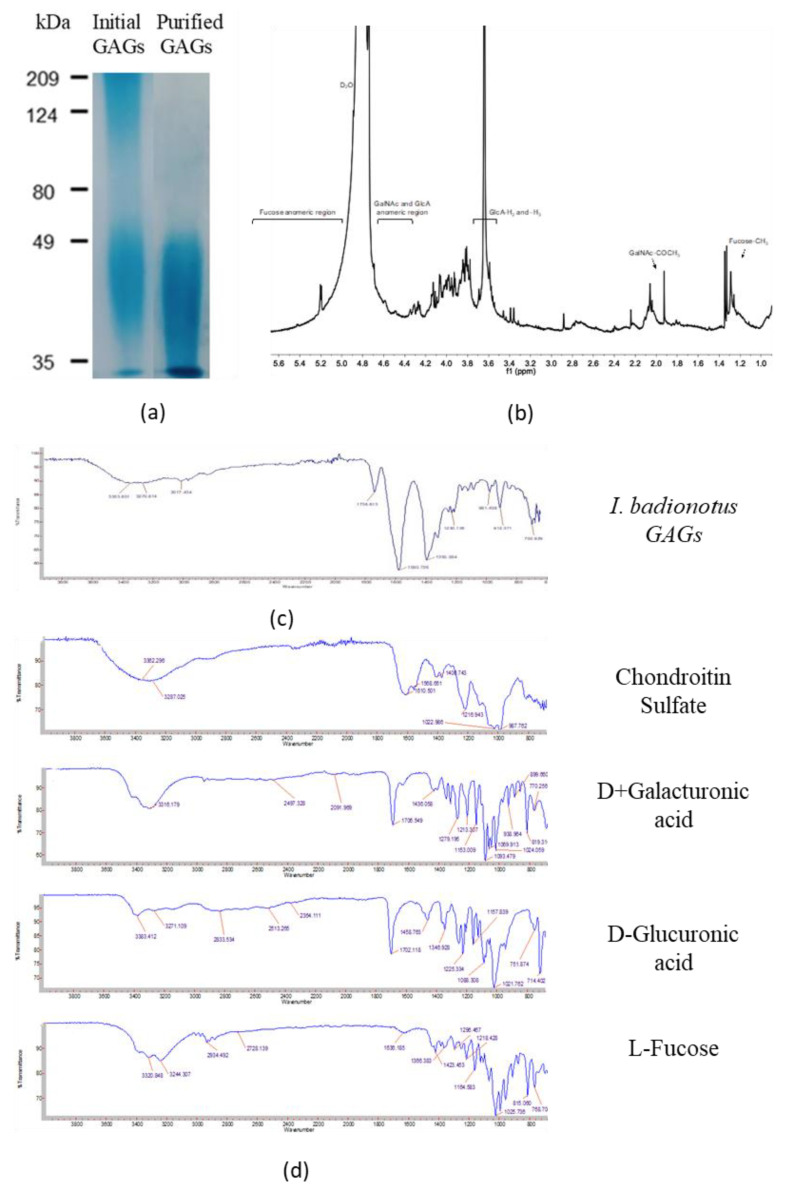
GAGs preparation isolated from *I. badionotus* body wall. (**a**) Alcian Blue-stained polyacrylamide gel electrophoretogram of initial and purified GAGs from sea cucumber (*I. badionotus*). (**b**) 1H NMR spectra at 600 MHz of fucosylated chondroitin sulfate of sea cucumber (*I. badionatus*). (**c**) Infrared spectra of GAGs from sea cucumber (*I. badionotus*). (**d**) Overlay comparing GAGs to standards.

The preliminary GAGs fraction prepared using the basic method of Vieira et al. [10] was comprised mainly of fucosylated chondroitin sulfate (FCS) but also contained small amounts of impurities, including fucoidan. An additional ion-exchange chromatography was applied to the isolation procedure. This method produced a glycosaminoglycan preparation comprised of fucose, N-acetyl-galactosamine, uronic acid and sulfates (Table 1). Polyacrylamide gel electrophoresis (Figure 2a), 1H NMR (Figure 2b; Supplemental Figure S2a) and infrared spectral analysis (Figure 2c,d, Supplemental Figure S2b) indicated the GAGS preparation to be fucosylated chondroitin sulfate (FCS). The sample appeared to be free of fucoidan and other contaminants. However, their presence in trace amounts in the GAGs preparation cannot be completely discounted.

The bioactivity in vitro of the purified FCS was confirmed since it was found to inhibit a TPA-mediated expansion in Interferon gamma (IFNγ)-positive HaCaT cells at levels of around 3 μg FCS/mL (Figure 1b). At much higher concentrations, FCS was cytotoxic (IC50, 80 μg/mL) in a human breast-derived fibroblast cell line (Supplemental Figure S1b).

### 3.3. FCS Exerts Anti-Inflammatory Activity In Vivo

**Figure 3:**
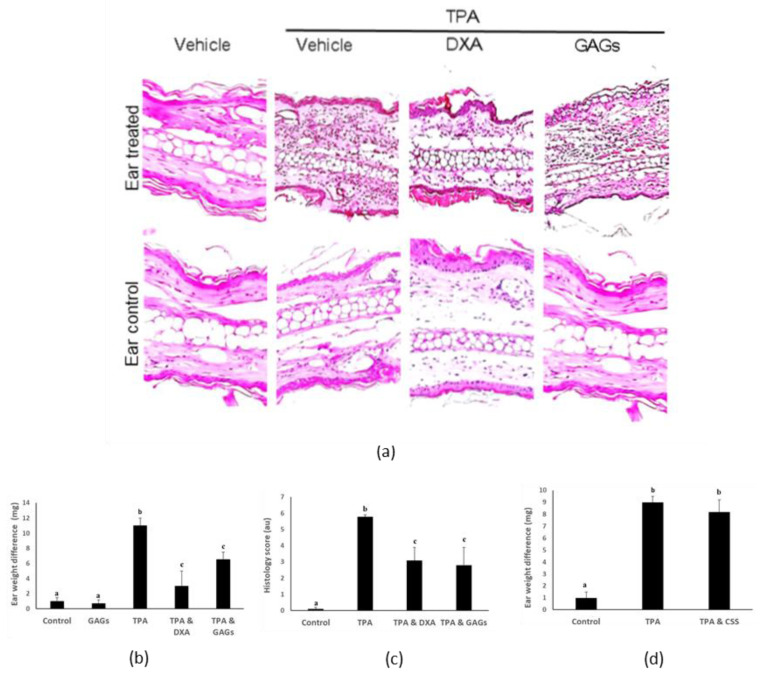
GAGs from sea cucumber (*I. badionotus*) body wall exert anti-inflammatory effects in a mouse ear inflammation model. Mouse ears exposed to vehicle (control, left ear), or inflammatory agent TPA alone, or TPA followed by dexamethasone (DXA) or GAGs (right ear). (**a**) Representative photomicrographs of transverse section of mouse ears sensitized with TPA or TPA and test substance, stained with haematoxylin-eosin (magnification 10X). Images are representative of five mice with similar results. (**b**) Differences in ear weights (right–left) after treatment with TPA or TPA and test substance; *n* ≥ 5 per group. (**c**) Histological score for ear micrographs in (**a**); values are the mean ± SEM, *n* = 3 per group. (**d**) Differences in ear weights (right–left) after treatment with TPA or TPA and shark cartilage (CSS); *n* ≥ 3 per group. Values with distinct superscripts differ significantly from each other (*p* ≤ 0.05).

Given their potent anti-inflammatory activity against keratinocytes in vitro we evaluated the biological activity of the purified FCS in a mouse ear inflammation model. As reported previously [7], topical administration of 12-O-tetradecanoylphorbol-13-acetate [TPA] to mouse ear triggered inflammatory cell infiltration, edema and major histological disruption within six hours (Figure 3a–c). Co-treatment of the ear with FCS attenuated these inflammatory responses, as did dexamethasone (Figure 3a–c). In contrast, chondroitin sulfate from shark cartilage had little or no effect on TPA-induced inflammation (Figure 3d).

**Figure 4:**
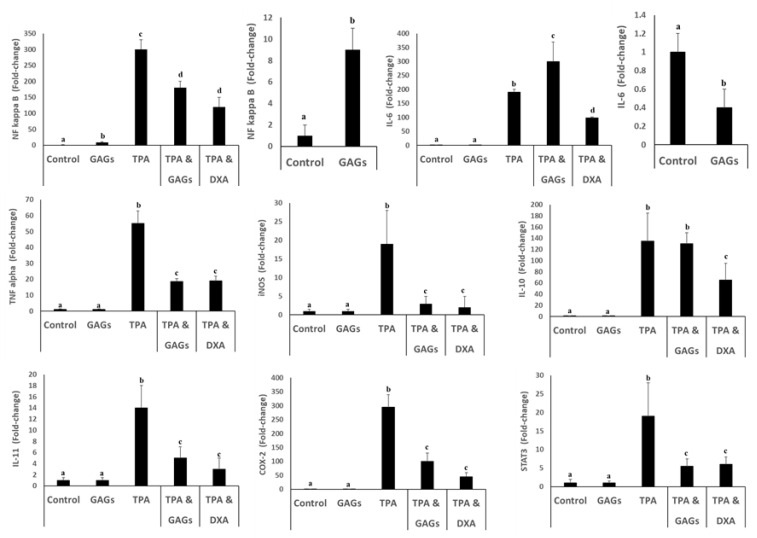
GAGs from sea cucumber (*I. badionotus*) target principal inflammation-related genes. Mouse ears were exposed to vehicle alone (left ear), GAGs, inflammatory agent TPA followed by GAGs, or TPA followed by dexamethasone (DXA) as an anti-inflammatory control (right ear). Genes NF kappa B (NF-ĸB), IL-6, TNF alpha (TNFα), iNOS, IL-10, IL-11, COX-2 and STAT3 were evaluated by qRT-PCR. Values (fold change) are the mean ± SD. Values with distinct superscripts differ significantly from each other (*p* < 0.05).

Expression of the TNFα, IL-6, NF-ĸB, iNOS, IL-10, IL-11, COX-2and STAT3 genes in mouse ear tissue increased following application of TPA (Figure 4). Co-administration of FCS suppressed these TPA-mediated responses, except for IL-6, which was increased further, and IL-10, which was unaffected (Figure 4). By contrast, TPA-mediated expression of all these genes was inhibited by co-treatment with dexamethasone (Figure 4). FCS alone had little or no effect on expression of inflammation-associated genes, except for NF-ĸB, which was slightly up-regulated and IL-6, which was marginally suppressed (Figure 4).

### 3.4. FCS from I. badionotus with Sulfate Levels of 4.5% or Above Have Similar Anti-Inflammatory Properties

**Figure 5:**
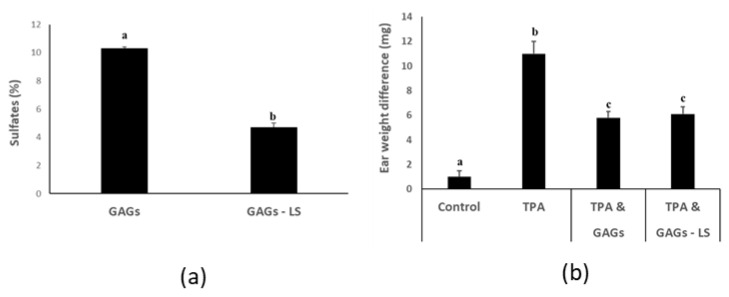
GAGs from sea cucumber (*I. badionotus*) with sulfate levels of 4.5% or above have similar anti-inflammatory properties. (**a**) The sulfate content of freshly-isolated GAGs used in analyses was quantified. The GAGs—LS were derived from the same sample but were reconstituted and lyophilized at least 3 times prior to sulfate analysis. (**b**) Differences in ear weights (right-left) in mouse ears exposed to vehicle alone (left ear), or inflammatory agent TPA followed by GAGs or GAGs—LS [low sulfate] (right ear). For each figure, *n* ≥ 4 per group and values with distinct superscripts differ significantly from each other (*p* ≤ 0.05).

During our studies we noted that sulfation of the purified FCS from *I. badionotus* steadily reduced when the preparation was repeatedly reconstituted and lyophilized (Figure 5a). Freshly prepared GAGs were tested alongside a sulfate-depleted sample in the mouse ear inflammation assay. Surprisingly, we found that the sulfate-depleted GAGs samples exhibited the same potent anti-inflammatory activity as did the freshly prepared ones (Figure 5b). This may indicate that sulfate levels of approximately 4.5% (*w*/*w*) may be adequate to maximize the anti-inflammatory actions of FCS from *I. badionotus*. 

### 3.5. Orally-Administered FCS from I. badionotus Ameliorate DSS-Colitis in Mice

**Figure 6:**
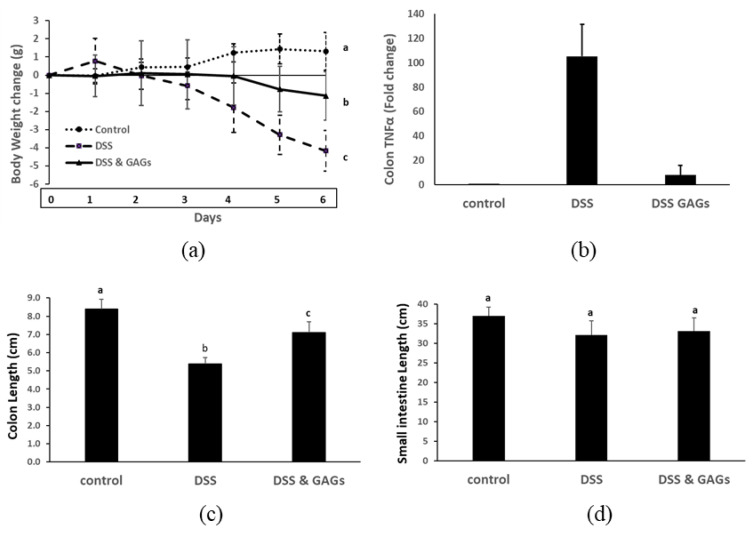
Orally-administered GAGs from sea cucumber (*I. badionotus*) body wall exert anti-inflammatory effects on Dextran sodium Sulfate (DSS)-colitis in mice. DSS (25 g/L in drinking water) was offered mice for four days, followed by water alone for two days. A proportion of the mice were dosed daily with GAGS (80 mg/kg body weight (BW)/d) for one day prior to and during the experimental period. (**a**) Weight change in mice dosed with DSS ± co-treatment with GAGs. (**b**) Colonic TNFα gene expression in mice dosed with DSS ± co-treatment with GAGs. (**c**) Colon lengths in mice dosed with DSS ± co-treatment with GAGs. (**d**) Small intestine lengths in mice dosed with DSS ± co-treatment with GAGs. For each figure, *n* ≥ 4 per group and values with distinct superscripts differ significantly from each other (*p* ≤ 0.05).

Having established that FCS was anti-inflammatory in the ear inflammation model, we then did a pilot study evaluating its ability to protect against acute intestinal inflammation in a DSS-colitis mouse model [20,21]. As expected, mice administered 2.5% DSS in their drinking water for four days followed by water alone for two days lost body weight (Figure 6a), the expression of TNFα gene in their colonic tissue was greatly up-regulated (Figure 6b) and their colons were significantly shortened (Figure 6c, Supplemental Figure S2). The actions of DSS on the intestine were confined to the lower bowel, meaning the small intestine was unaffected by this treatment (Figure 6d). 

Oral administration of FCS immediately prior to and during the experimental period counteracted the effects of DSS on mice (Figure 6a–c; Supplemental Figure S3). In other words, co-treatment with FCS attenuated the body weight loss, expression of colonic TNFα gene and colon shortening caused by DSS.

## 4. Discussion

Previous studies established that lyophilized sea cucumber (*Isostichopus badionotus*) from the Yucatan Peninsula have potent anti-inflammatory actions when tested in various animal models [7]. We have now shown that these protective effects are multi-factorial, and are largely mediated by fucosylated chondroitin sulfate (FCS), a principal component of the body wall. This natural occurring bioactive component limited the responses of HaCat (keratinocyte) cells to 12-O-tetradecanoylphorbol-13-acetate (TPA), modulated expression of critical genes and attenuated inflammation and tissue damage caused by TPA in mouse ear and mitigated colonic colitis caused by dextran sodium sulfate (DSS) in a pilot study in mice. 

FCS from *I. badionotus* ameliorates hyperlipidaemia and metabolic syndromes in mice [22,23]. Given our original finding that *I. badionotus* meal was both hypolipidemic and anti-inflammatory in animal models [6,7], the present observations that *I. badionotus* FCS suppressed skin and intestinal inflammation is not inconsistent with the findings of Li et al. [22,23], and indicates that *I. badionotus* FCS has a broad spectrum of health-promoting actions in vivo. 

Fucoidans from sea cucumber, including those of *I. badionotus*, are also known to have anti-inflammatory, hypolipidemic and health-promoting properties in vivo [24,25,26]. As we found in our preliminary studies, small amounts of fucoidan can co-extract with glycosaminoglycans in the Vieira et al. [10] isolation procedure. However, introduction of an ion exchange chromatography step in the purification process significantly reduced the residual amounts of fucoidan in the final FCS preparation. The fucoidan doses necessary to ameliorate experimental hyperlipidaemia and metabolic syndrome in vivo are similar [24,25,26] to those required for FCS to treat the same experimental disorders [22,23]. Given that our FCS preparation contained at most only trace amounts of fucoidan, it is unlikely that fucoidan made a significant contribution to the potent anti-inflammatory actions of our *I. badionotus* FCS preparation. 

The cytotoxicity of *I. badionotus* FCS for cells in culture was higher (IC50, 80 μg/mL) than previously reported [27]. However, only low levels (3 μg/mL) of FCS were needed to suppress TPA-induced responses in HaCaT cells, and mouse splenocytes in vitro; indeed these were at least thirteen-to fifteen-fold less than needed to cause any significant adverse effects on human fibroblast viability in vitro. Therefore, cytotoxicity did not influence the anti-inflammatory properties of *I. badionotus* FCS.

Sulfation levels, sulfate pattern (2,4-O-di-sulphation) and alpha-L-fucosyl branched units are considered essential factors in FCS bioactivity, particularly its anticoagulative and anti-thrombotic activities [28,29,30,31]. However, we found that sulfate concentrations over 4.5% were of no apparent advantage for the anti-inflammatory actions of *I. badionotus* FCS. This level of sulfation may therefore be the threshold required for maximal bioactivity.

The composition, structure and bioactivity of sea cucumber FCS vary considerably between species and even within the same species depending on the exact location and environment of the capture site [27,28,32]. It is, therefore, essential to establish that FCS derived from a new source has the required therapeutic properties for practical clinical use. The results of this study demonstrate that FCS from *I. badionotus* captured off the Yucatan Peninsula has excellent potential for use in clinical treatment or management of inflammatory disorders.

TPA activates the nuclear factor-B molecular signaling pathway. This, in turn, initiates the expression of pro-inflammatory genes, such as TNFα and COX-2, as well as a range of cell survival factors essential for the preservation of cellular integrity and subsequent down-regulation of acute inflammatory responses [28,33,34,35]. FCS limited inflammation and tissue damage in TPA-treated mouse ears by down-regulation of NF-ĸB and down-stream genes such as COX-2 and TNFα. *I. badionotus* FCS’s mode of action is unknown, but reports for some sea cucumbers indicate that their constituent glycosaminoglycans can act on the NF-ĸB-pathway by blocking the translocation of active NF-ĸB [RelA(p65)/p50] from the cytoplasm to the nucleus, thereby preventing activation of downstream genes [36,37]. This action would be consistent with the gene changes observed in TPA/FCS-treated ear samples except for the slight upregulation of NF-ĸB caused by FCS alone. However, if FCS prevented even basal levels of active NF-ĸB from reaching the nucleus, the small FCS-linked increase in NF-ĸB expression may have been a compensatory response aimed at overcoming the loss of molecular signaling. IL-6 expression is highly dependent on NF-ĸB [38] so the reduced expression of IL-6 in FCS-treated ear may also be a result of the failure of active NF-ĸB to reach or accumulate in the nucleus. 

DSS activates NF-ĸB in colonic epithelium and up-regulates an array of pro-inflammatory genes. These actions cause chronic colonic inflammation, tissue disruption, a characteristic shortening of the colon and rapid loss of body weight [20,39]. Oral administration of *I. badionotus* FCS (80 mg/kg BW/d) attenuated the harmful effects of DSS on the colon and limited the loss of body weight caused by DSS. As in the ear inflammation assay, this amelioration of DSS colitis may have been due to the direct inhibitory effects of FCS upon NF-ĸB in epithelial cells leading to a diminution in colonic inflammation. 

FCS may also have had indirect protective actions. DSS disrupts the intestine microbiota, leading to loss of protective and short-chain fatty acid (SCFA)-producing bacteria and a concomitant expansion in the populations of opportunistic pathobionts [40,41,42]. In previous studies, FCS partially ameliorated intestinal dysbiosis associated with experimental metabolic syndromes [22,41]. *I. badionotus* FCS could therefore have similar effects on DSS-induced dysbiosis. It may reduce the levels of opportunistic pathogens and promote an expansion in the numbers of SCFA-producers or protective bacteria which produce compounds that inhibit activation, release, or translocation of NF-ĸB [20,39,41,43]. These actions would limit the severity of epithelial inflammation and gut damage caused by DSS. 

*I. badionotus* body wall has potent anti-inflammatory actions in vivo [6,7]. Here we show that these properties are due, at least in part, to FCS, one of its major extractable components. However, in agreement with reports for other species [1,2], our in vitro data also indicates that other body wall constituents have anti-inflammatory properties. It remains to be established whether the naturally occurring bioactive components in *I. badionotus* body wall act additively or synergistically to provide its overall anti-inflammatory effect.

## 5. Conclusions

*I. badionotus* body wall contains several components with anti-inflammatory properties. They may act additively or synergistically to give maximal activity. Nonetheless, the present pre-clinical studies have established that *I. badionotus* FCS has pharmacologically promising anti-inflammatory activity on its own. Given the immense need for novel anti-inflammatory products of a known and predictable mode of action, robust clinical trials are needed to establish if *I. badionotus* FCS has similar demonstrable and repeatable health benefits for humans.

## Figures and Tables

**Table 1:** Chemical composition (*w*/*w*, %) of GAGs extracted from *Isostichopus badionotus* from the Yucatan Peninsula compared to a non-fucosylated chondroitin sulfate from shark cartilage (CSS).

	GAGs	CSS
Total CHO	24.77 ± 2.5	25.36 ± 0.06
Fucose	6.40 ± 0.54	ND
Uronic acid	8.55 ± 0.48	20.6 ± 0.04
Sulfates	11.12 ± 0.60	15.06 ± 0.001
Soluble proteins	4.15 ± 0.01	5.01 ± 0.001

Results are the average of three independent determinations ± SD. CHO = carbohydrates; ND = not detected.

## Supplementary Materials

The following are available online at https://www.mdpi.com/2072-6643/12/6/1698/s1, Figure S1: (a) Crude GAGs from the body wall of *I. badionotus* inhibit 12-O-tetradecanoylphorbol-13-acetate (TPA)-induced proliferation of mouse splenocytes. (b) High concentrations of purified GAGs reduce the viability of a human breast-derived fibroblast cell line. N ≥ 4 per group and values with distinct superscripts differ significantly from each other (*p* ≤ 0.05). Figure S2: (a) 1H NMR spectra at 600 MHz of fucosylated chondroitin sulfate of sea cucumber (*I. badionatus*). (b) Infrared spectra of GAGs from sea cucumber (*I. badionotus*). Figure S3: Representative colons from mice treated with DSS and *I. badionotus* GAGs (a), or DSS (b) and untreated controls (c).
