# Supplementary material for: A Glycosaminoglycan-Rich Fraction from Sea Cucumber Isostichopus badionotus Has Potent Anti-Inflammatory Properties In Vitro and In Vivo
Source: Nutrients. 2020 Jun 6;12(6):1698. doi: 10.3390/nu12061698 (PMC7352476; doi:10.3390/nu12061698)
Supplement: Supplementary file 1 [file nutrients-12-01698-s001.pdf]

Online Supplementary Materials

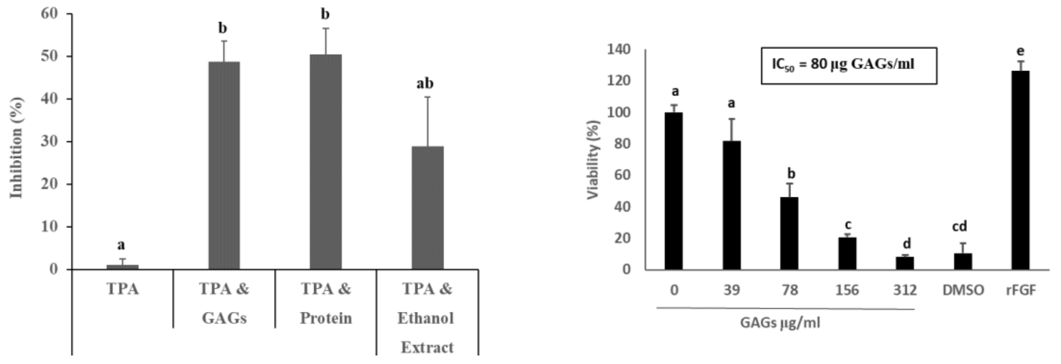

(a) (b)

Supplemental Figure S1. Crude GAGs from the body wall of*Ibadionotus* inhibit 12-O-tetradecanoylphorbol-13-acetate (TPA)-induced proliferation of mouse splenocytes (a). High concentrations of purified GAGs reduce the viability of a human breast-derived fibroblast cell line (b). N≥4 per group and values with distinct superscripts differ significantly from each other (p≤0.05).

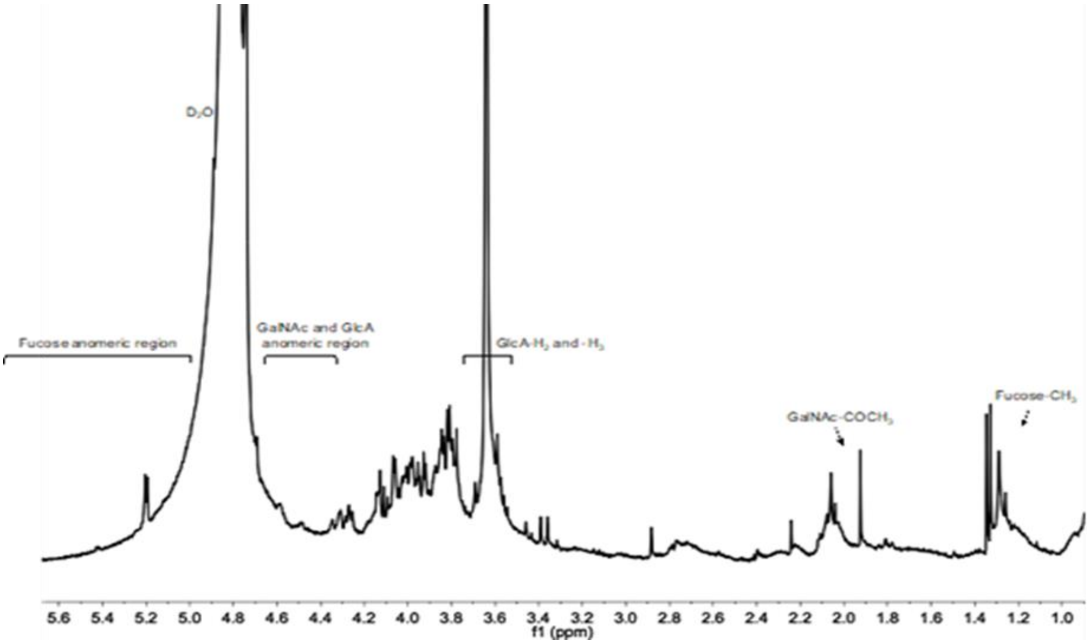

(a)

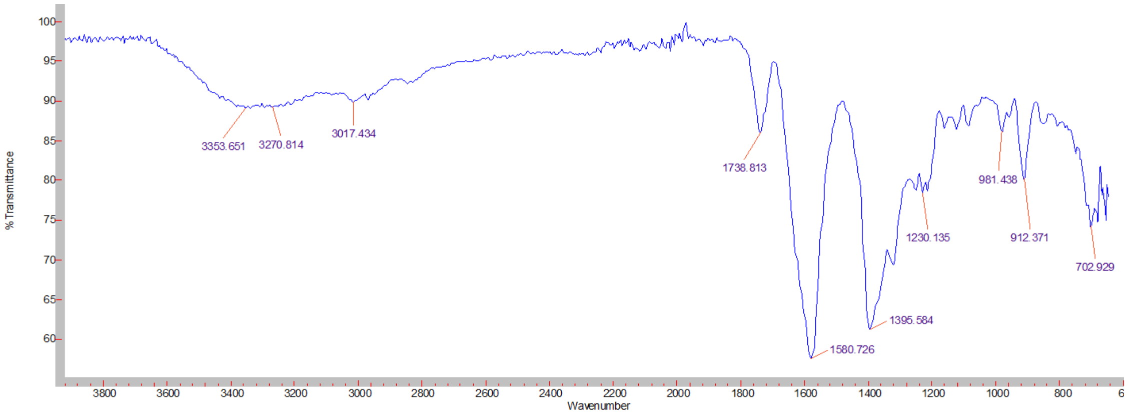

(b)

Supplemental Figure S2. **GAGs preparation isolated from body wall of *I. badiono*tus**
a) 1H NMR spectra at 600 MHz of fucosylated chondroitin sulphate of sea cucumber (I. *badionatus*)
b) Infrared spectra of GAGs from sea cucumber (*I. badionotus*).

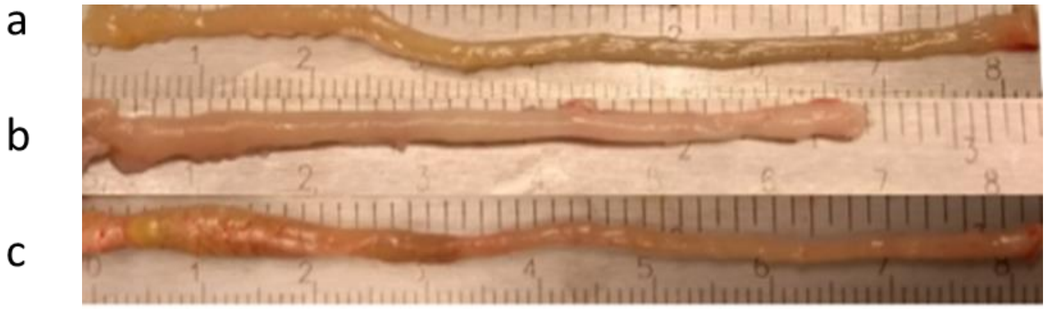

Supplemental Figure S3. Representative colons from mice treated with DSS & *I badionotus* GAGs (a), or DSS (b) and untreated controls (c).
